# A pharmacogenetic pilot study reveals *MTHFR*, *DRD3*, and *MDR1* polymorphisms as biomarker candidates for slow atorvastatin metabolizers

**DOI:** 10.1186/s12885-016-2062-2

**Published:** 2016-02-08

**Authors:** Rafael B. R. León-Cachón, Jorge A. Ascacio-Martínez, María E. Gamino-Peña, Ricardo M. Cerda-Flores, Irene Meester, Hugo L. Gallardo-Blanco, Magdalena Gómez-Silva, Everardo Piñeyro-Garza, Hugo A. Barrera-Saldaña

**Affiliations:** Centro de Diagnóstico Molecular y Medicina Personalizada, Departamento de Ciencias Básicas, División Ciencias de la Salud, Universidad de Monterrey, San Pedro Garza García, NL México; Departamento de Bioquímica y Medicina Molecular, Facultad de Medicina, Universidad Autónoma de Nuevo León, Monterrey, NL México; Ipharma S.A., Monterrey, NL México; Facultad de Enfermería, Universidad Autónoma de Nuevo León, Monterrey, NL México; Departamento de Genética, Universidad Autónoma de Nuevo León, Monterrey, NL México; Vitagénesis S.A., Monterrey, NL México

**Keywords:** Atorvastatin, Genotype phenotype association, Predictive genetic testing

## Abstract

**Background:**

The genetic variation underlying atorvastatin (ATV) pharmacokinetics was evaluated in a Mexican population. Aims of this study were: 1) to reveal the frequency of 87 polymorphisms in 36 genes related to drug metabolism in healthy Mexican volunteers, 2) to evaluate the impact of these polymorphisms on ATV pharmacokinetics, 3) to classify the ATV metabolic phenotypes of healthy volunteers, and 4) to investigate a possible association between genotypes and metabolizer phenotypes.

**Methods:**

A pharmacokinetic study of ATV (single 80-mg dose) was conducted in 60 healthy male volunteers. ATV plasma concentrations were measured by high-performance liquid chromatography mass spectrometry. Pharmacokinetic parameters were calculated by the non-compartmental method. The polymorphisms were determined with the PHARMAchip® microarray and the TaqMan® probes genotyping assay.

**Results:**

Three metabolic phenotypes were found in our population: slow, normal, and rapid. Six gene polymorphisms were found to have a significant effect on ATV pharmacokinetics: *MTHFR* (rs1801133), *DRD3* (rs6280), *GSTM3* (rs1799735), *TNFα* (rs1800629), *MDR1* (rs1045642), and *SLCO1B1* (rs4149056). The combination of *MTHFR*, *DRD3* and *MDR1* polymorphisms associated with a slow ATV metabolizer phenotype.

**Conclusion:**

Further studies using a genetic preselection method and a larger population are needed to confirm these polymorphisms as predictive biomarkers for ATV slow metabolizers.

**Trial registration:**

Australian New Zealand Clinical Trials Registry: ACTRN12614000851662, date registered: August 8, 2014.

**Electronic supplementary material:**

The online version of this article (doi:10.1186/s12885-016-2062-2) contains supplementary material, which is available to authorized users.

## Background

The drug atorvastatin (ATV) is widely prescribed to treat hypercholesterolemia, which is a predisposing factor for developing atherosclerosis. ATV, like all statins, acts by inhibiting 3-hydroxy-3-methylglutaryl-coenzyme A reductase, an essential enzyme in cholesterol biosynthesis. ATV reduces the risk of atherosclerosis by lowering the levels of low-density lipoprotein-bound cholesterol [1]. ATV is administered orally, as a calcium salt (acid form), at a dose that ranges between 10 and 80 mg/day. Once ATV is ingested, several enzymes participate in its metabolism. The enzymes encoded by *CYP3A4* and *CYP3A5* are the most important ones [2]. These enzymes transform ATV first to its lactone form and subsequently into 2 pharmacologically active metabolites (2-hydroxy-ATV and 4-hydroxy-ATV) [3, 4]. Next, ATV and its secondary metabolites are glucuronidated by uridine diphosphoglucuronosyltransferases (encoded by *UGT1A1* and *UGT1A3*) [5]. Apart from metabolic enzymes, carrier proteins are involved in ATV metabolism, such as: 1) P-glycoprotein, also known as multidrug resistance protein 1 (MDR1), product of *ABCB1*, 2) the organic anion-transporting polypeptides (OATP1B1 and OATP1B3) encoded by *SCLO1B1* and *SCLO1B3*, respectively [6–8], and 3) the breast cancer resistance protein (BCRP2), product of *ABCG2* [9, 10].

Drug performance is evaluated on two main areas: pharmacokinetics and pharmacodynamics. The former focuses primarily on the evaluation of absorption, distribution, metabolism, and excretion processes (ADME) of a drug; consequently, the variation in genes responsible for this process contributes to interindividual variability [11]. On the other hand, pharmacodynamics evaluates biochemical and physiological effects, as well as the mechanisms of action of a drug, *i.e*. it focuses on the drug response [12]. The pharmacokinetics of ATV displays high interindividual variability of up to 30 % [13, 14]. This pharmacokinetic discrepancy reveals variations in the ADME processes as the drug passes through the human body [12]. Factors such as age, gender, ethnicity, and genetic variability are involved in such interindividual differences [15, 16]. Genetic factors are responsible for about 15 to 30 % of the interindividual variation in metabolism, and thus in the response of each patient to certain classes of drugs. However, for some drugs this percentage can increase to up to 95 % [17]. These genetic factors are mainly due to variations in short tandem repeats, copy number variations, insertions and deletions, and single-nucleotide polymorphisms (SNPs). The latter is the most common source of variation [18–20]. The presence of these polymorphisms in genes involved in the metabolism of ATV may explain its pharmacokinetic variability [17], since the frequency and consequences also vary between different populations [21]. The aims of this study were: 1) to reveal the frequency of 87 polymorphisms in 36 genes related to drug metabolism in healthy volunteers, 2) to evaluate the impact of these polymorphisms on ATV pharmacokinetics, 3) to classify the metabolic phenotypes for ATV, and 4) to investigate a possible association between genotypes and phenotypes.

## Methods

### Design

A randomized pilot study was carried out in 60 healthy Mexican volunteers to determine ATV pharmacokinetic parameters. A single dose of 80 mg ATV was administered. The clinical study complied with Good Clinical Practice standards, the guidelines of the Declarations of Helsinki and Tokyo, and the Mexican regulations on Bioavailability and Bioequivalence Studies (NOM-177-SSA1-1998) [22]. Furthermore, the protocol was approved by the Research and Ethics Committee of the pharmacokinetic study center, Ipharma S.A. (Monterrey, Mexico). The clinical study has been registered at the Australian New Zealand Clinical Trials Registry (registration number: ACTRN12614000851662).

### Study population

Sixty four healthy male candidates from northeastern Mexico were recruited and a written informed consent was obtained. Inclusion criteria were: non-smoker, 18-to-45-year old, weight ≥ 50 kg, body mass index (BMI) of 20–26 kg/m^2^, availability for completing the study, being healthy. Since ATV is classified as a pregnancy category X drug, only males were considered for the study. Candidates were excluded for: any abnormal lab result, significant personal or family medical history of angioedema o allergies, the existence of concurrent disease, use of prescription or over-the-counter medication or alcohol before enrollment, history of smoking, alcohol or drug abuse, and incompliance or non-willingness to complete the study. Four candidates were excluded because of the consumption of alcohol and/or substances or an altered blood pressure. The health status of the volunteers was confirmed by a medical history, a physical examination, an electrocardiogram (ECG), laboratory tests (blood count, blood chemistry, liver function tests, and urinalysis), and seronegativity for human immunodeficiency virus and hepatitis B and C viruses.

### Drug administration and sampling

After an overnight fast at the study center (Ipharma, S. A.), each subject was given a single dose of 80 mg of ATV-coated tablets (Pfizer Pharmaceuticals LLC, Caguas Site, Caguas, PR). The volunteers were under direct medical supervision at the study site. Venous blood (4 mL) was collected in K_2_EDTA-coated Vacutainers^TM^ (BD Diagnostics, Franklin Lakes, NJ, US), before ATV administration (time 0), and at the following time points after drug administration: 0.25, 0.5, 0.75, 1, 1.5, 2, 2.5, 3, 3.5, 4, 5, 6, 8, 12, 24, 36 and 48 h. Plasma was separated by centrifugation (15 min at 1600 g at 4 °C) and stored in cryovials at −80 °C until analysis, using a method validated by Ipharma S. A. [3, 23, 24].

### Pharmacogenetic tests

Leukocytes were obtained from the buffy coat, and genomic DNA was extracted by the alkaline lysis method [25]. Seven multiplex polymerase chain reactions (PCRs) amplified the desired gene regions, following a validated protocol [26]. Screening for gene polymorphisms was performed using the PHARMAchip® microarray (Progenika, Derio, ES). This pharmacogenetic genotyping device detects, with a 99.9 % specificity and sensitivity, 85 gene polymorphisms in 34 genes involved in drug metabolism and response, including those encoding cytochrome P450 enzymes, phase II metabolism enzymes, receptors, and transporters. Amplified products were fractionated with DNAse according to a validated protocol [26], followed by fluorescent labeling and hybridization of the microarray, in an automated TECAN HS4800PRO platform (Ventana Medical Systems Inc., Tucson, AZ, US). The hybridization pattern was revealed using the Innoscan 710 scanner (Innopsys S.A., Carbonne, FR). Polymorphic variants were determined using PHARMAchip software V.3.2.9 [26]. Two additional polymorphisms not included in the PHARMAchip, rs2231142 (C__15854163_70) and rs4149056 (C__30633906_10), in *ABCG2* and *SLCO1B1* respectively, were included in the study and analyzed by Real-Time PCR system using validated Genotyping Assays (Applied Biosystems, Foster City, CA, US) according to the manufacturer’s instructions. Typed polymorphisms were only includedin subsequent association studies after having passed three quality control tests: the genotype call rate (>0.90 completeness to obtain 99.8 % accuracy), the Hardy-Weinberg equilibrium (HWE) test (*P*-value > 0.05), and the minor allele frequency (MAF) criterion (>0.01).

### Determination of ATV calcium in plasma

Proteins were eliminated from the plasma samples by adding 4 volumes of acetonitrile to 100-μL samples, vortexing (70 rpm, 4 min.), and precipitating by centrifugation (9600 g, 10 min., 10 °C). Protein-free supernatant (300 μL) was recovered and 5-μL samples were injected into an Agilent 1100 high-performance liquid chromatographer (HPLC; equipped with an autosampler and a binary pump), which was connected to an Agilent 6410 tandem mass spectrometer (MS/MS) with a triple quadrupole detector (Agilent Technologies, Santa Clara, CA, US) to measure ATV calcium levels. A C_18_ pre-column and a Synergi^TM^ Fusion-RP column (4 μm, 80 Å, 50 × 2 mm; Phenomenex, Torrance, CA, US) formed the solid phase, whereas the mobile phase consisted of 0.03 % formic acid/70 % acetonitrile in analytical grade water. The column temperature was 40 °C, the flow rate 0.4 mL/min, and the auto-sampler temperature 20 °C. The detection system used an ESI MS/MS precursor ion (+) 559.3 m/z and a product ion (+) 440.3 m/z. Under these conditions, interday linearity was assessed by performing calibration curves from 0.5 to 100 ng/mL (0.5, 2.5, 5, 10, 25, 50 and 100); intraday quality control was evaluated by using eight ATV control samples of 1.7, 7.5, 35, and 75 ng/mL each.

### Pharmacokinetic analysis

WinNonlin® professional software V.5.3 (Pharsight Corp., Mountain View, CA, US) was used for pharmacokinetic analysis. The maximum plasma concentration (C_max_) and the time to reach C_max_ (T_max_) were calculated from the observed concentration-time data in plasma. Pharmacokinetic parameters were estimated with the non-compartmental method after oral administration of a single dose of ATV and were as follows: 1) the area under the plasma concentration-time curve from time 0 to the time of the last measurement (AUC_0-t_), calculated using the logarithmic-linear trapezoidal rule, 2) the area under the curve from time 0 to the time extrapolated to infinity (AUC_0-∞_), 3) the apparent clearance of the fraction dose absorbed (Cl/F), 4) the elimination rate constant in the terminal phase (Ke), and 5) the half-life in the terminal phase of the drug (T_1/2_).

### Statistical analyses

For sample size calculation, it was assumed that the coefficient of variation (CV) was 45 % for the C_max_ and AUC of ATV. Considering a significance level of 5 %, a minimum power of 80 %, an Ω of 0.25, and a confidence interval of 90 %, a sample size of 58 would suffice. The metabolizer phenotypes classification was made using a multivariate analysis of the combined pharmacokinetics parameters C_max_ and AUC_0-t_. To minimize the effect of scale differences, before calculating the distance matrix, these variables were standardized. Next, the individual values of C_max_ and AUC_0-t_ were subjected to hierarchical cluster analysis (HCA) using the Ward linkage method and the interindividual Manhattan distances were computed. The standardization, HCA, and the hierarchical clustering dendogram were made using Minitab 16 demo software (Minitab Inc., State College, PA, US) [27]. We identified the participants of each cluster and calculated the geometric means of all pharmacokinetic parameters of each cluster. According to the geometric means of the pharmacokinetic parameters of the clusters they were classified into metabolizer phenotypes. Next, one-way ANOVA and the Kruskal-Wallis H test were used to validate the classification model. The HWE was determined by comparing the genotype frequencies with the expected values using the maximum likelihood method [28]. To detect significant differences between 2 groups, Student’s *t*-test or the Mann Whitney *U* test were used for parametric and non-parametric distributions, respectively. Differences between more than 2 groups were assessed by one-way ANOVA or the Kruskal-Wallis H test for parametric and non-parametric distributions, respectively. Post hoc tests (LSD and Tamhane’s T2) were used for pairwise comparisons. To evaluate the contribution of genetic factors to the variability of the pharmacokinetic parameters linear regression analysis was done. Possible associations between genotypes or genotype combinations and phenotypes were assesed using contingency tables *Χ*^2^ statistics and Fisher’s exact tests. The linear regression analysis and association studies were performed under three different models (dominant, recessive, and additive). Odds ratios were estimated with 95 % confidence intervals. The model for prediction was confirmed using stepwise multiple linear regression analysis. Aforementioned analyses were performed with SPSS for Windows, V.20 (IBM Corp., Armonk, NY, US). All *P*-values were two-tailed. The corrected *P* (Pc) values were adjusted according to Bonferroni’s correction for multiple comparisons and the Benajmini-Hochberg procedure was applied to exclude spurious associations [29]. A *P*-value ≤ 0.05 was considered statistically significant.

## Results

### Study population

Sixty male subjects completed the study. Volunteers were of mestizo descent, most of them students (73 %) from the state of Nuevo Leon (83 %). Other demographic characteristics did not display significant variability (Table 1).

**Table 1:** Demographic data of volunteers

	Gender	BMI (kg/m^2^)	BS (m^2^)	Age (years)	Weight (kg)	Height (m)	n
Mean	M	23.43	1.84	24.01	70.58	1.73	60
SD		1.64	0.131	4.35	8.24	0.065	

*BMI* body mass index; *BS* body surface area; *M* male. *SD* Standard Deviation. Data shown as mean (± SD)

### ATV pharmacokinetics

Despite controlling physiological and environmental conditions the pharmacokinetic parameters were highly variable (Table 2). The geometric mean ± SD for the pharmacokinetic parameters obtained were: C_max_ = 41.44 ± 23.35 ng/mL, AUC_0-t_ = 141.88 ± 86.78 ng/mL*h, AUC_0-∞_ = 157.12 ± 87.24 ng/mL*hr, Cl/F = 509.20 ± 265.57 L/h, T_1/2_ = 9.81 ± 6.58 h, and the Ke = 0.071 ± 0.035.

**Table 2:** Pharmacokinetic parameters according to metabolizer phenotype

	All subjects	Phenotype		
Parameters		Slow	Normal	Rapid
N	60	18	25	17
C_max_ (ng/mL)	41.44 ± 23.35	75.39 ± 15.74*	40.48 ± 6.37*	22.11 ± 8.15*
AUC_0-t_ (ng/mL*h)	141.88 ± 86.78	218.14 ± 101.90*	152.69 ± 48.83*	80.78 ± 27.24*
AUC_0-∞_ (ng/mL*h)	157.12 ± 87.24	231.55 ± 100.02*	166.35 ± 55.59*	95.81 ± 28.38*
Cl/F (L/h)	509.20 ± 265.57	345.49 ± 119.94*	480.93 ± 152.07*	834.97 ± 249.52*
T_1/2_ (h)	9.81 ± 6.58	9.64 ± 6.18	10.76 ± 7.54	8.71 ± 5.43
Ke	0.071 ± 0.035	0.072 ± 0.041*	0.064 ± 0.030*	*0.080 ± 0.035

Data shown as geometric mean (± Standard Deviation)

*statistically significant (*P* ≤ 0.016)

### Classification of metabolizer phenotypes

**Fig. 1:**
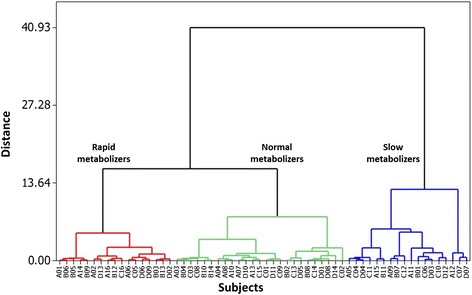
Classification of ATV metabolic phenotypes. A dendrogram generated with the Manhattan distance and Ward’s linkage method illustrates rapid metabolizers (*red*), normal metabolizers (*green*), and slow metabolizers (*blue*)

**Fig. 2:**
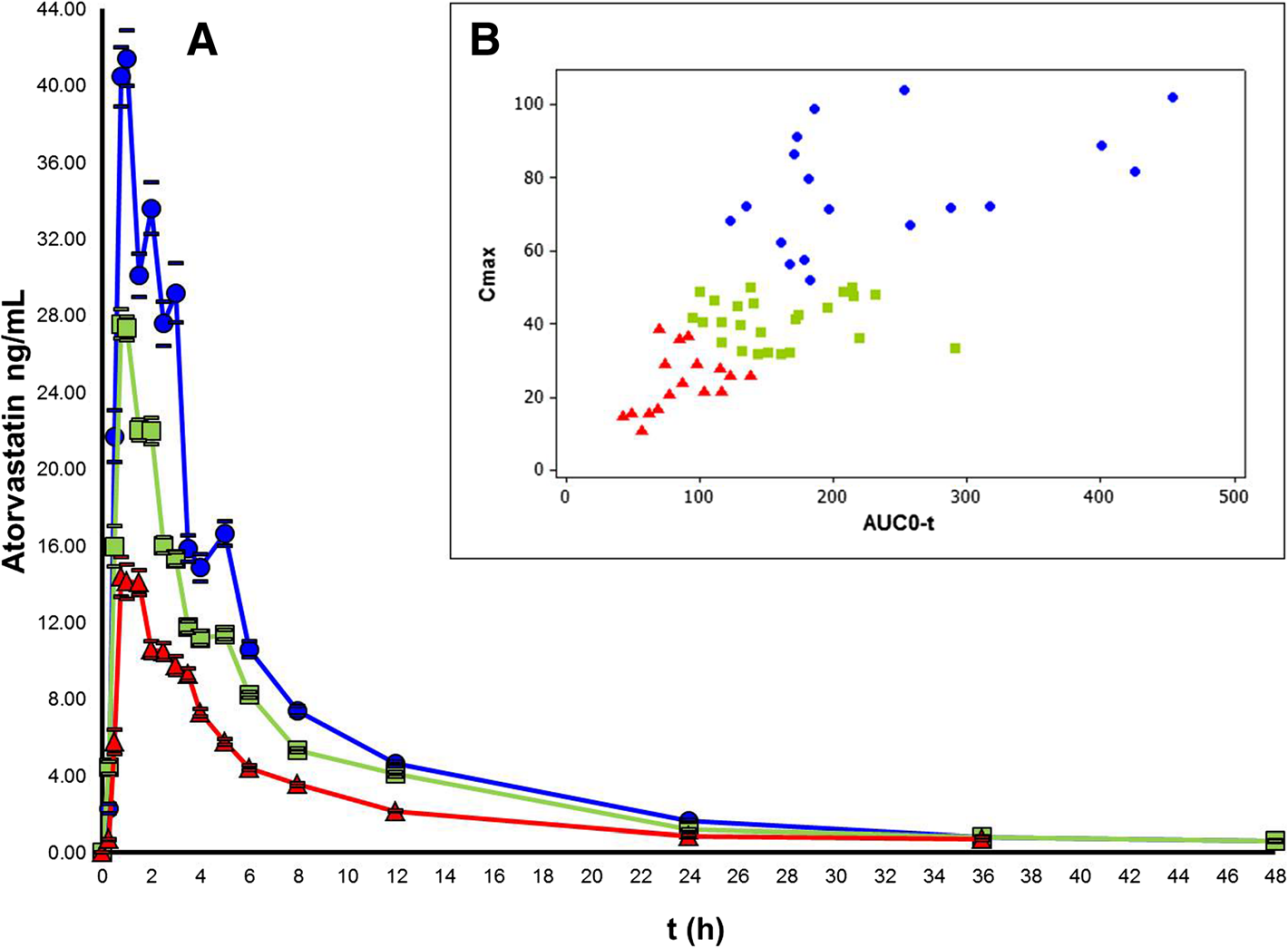
Distinctive pharmacokinetic profiles of rapid, normal and slow metabolizers. **a** Mean peak plasma ATV concentration-time curves after a single 80-mg dose of ATV of the three metabolizer phenotypes. Data shown are mean ± standard error (SE) concentrations. **b** Distributions of metabolizer phenotypes with regard to C_max_-AUC_0>-t_ values. For both **a** xand **b**: rapid metabolizers in *red*, normal in *green*, and slow ones in *blue*

C_max_ and AUC_0-t_ were used for HCA classification of pharmacokinetic profiles, because C_max_ tends to best reveal differences in pharmacokinetic profiles and AUC_0-t_ is considered to be the best parameter to evaluate a drug’s interindividual pharmacokinetic variation [30]. The HCA, based on centroid distance, revealed three main clusters. Which we identified as slow metabolizers (30.00 %), normal metabolizers (41.66 %), and rapid metabolizers (28.33 %), as shown in Fig. 1. The geometric means of the pharmacokinetics parameters were significantly different among the three clusters (*P* ≤ 0.016), except for T_1/2_ (Table 2). The mean concentration-time profile and the geometric mean pharmacokinetic parameters of ATV obtained for each metabolizer phenotype are shown in Fig. 2a and Table 2, respectively. We observed a > 9-fold difference in ATV pharmacokinetic paratmeters between the fastest metabolizer indivual (C_max_ = 10.94 ng/mL and AUC_0-t_ = 55.23 ng/mL*h) and the slowest metabolizer individual (C_max_ = 101.85 ng/mL and AUC_0-t_ = 454.41 ng/mL*h). The distribution of phenotypes regarding C_max_-AUC_0-t_ values, are presented in Fig. 2b.

### Pharmacogenetic tests

The allele and genotype frequencies of the gene polymorphisms with a potential impact on drug metabolism are presented in Additional file 1. The HWE applied to most of the gene polymorphisms, with the exception of the gene deletions of *GSTM1* and *GSTT1*, because the heterozygous variants were not detected [31]. The polymorphism rs1800896 in the *IL10* was not in HWE equilibrium. The polymorphisms in *CYP2D6*, *NAT2*, *TPMT*, and *TYMS* were below the call rate threshold of 0.9. The SNPs in *DPYD*, rs1799807 in *BCHE*, and rs28399504 and rs41291556 in *CYP2C19* had a MAF < 0.01. The aforementioned polymorphisms were excluded from subsequent analyses; a total of 30 SNPs remained for statistical analysis.

### Association between gene polymorphisms and ATV pharmacokinetics

The various pharmacokinetic parameters were affected differentially by the different genetic *loci*;*i.e*. a certain polymorphism had an effect on C_max_ but not on AUC_0-t_ or the other way round, while there were also polymorphisms that affected both and/or other parameters (Table 3). The effect of *MTHFR*-rs1801133 on C_max_ was statistically significant. Heterozygous variant (C/T) carriers and homozygous variant (T/T) carriers had lower C_max_ values compared to homozygous wild-type (C/C) carriers (*P* = 0.018 and 0.004, respectively). Carriers of the variant genotype (C/T or T/T) showed significant lower values of C_max_ (*P* = 0.006), AUC_0-t_ (*P* = 0.050) and AUC_0-∞_ (*P* = 0.044) but statistically significant higher values of Cl/F (*P* = 0.044) as compared to homozygous wild-type subjects (C/C). The genotypes resulting from the *DRD3*-rs6280 (Ser9Gly) polymorphism had a significant impact on ATV pharmacokinetics. First, the homozygous wild-type (C/C) carriers had lower T_1/2_ values (*P* = 0.003) and higher Ke values (*P* = 0.008) as compared to homozygous variant (T/T) carriers. Second, when comparing T/T with (C/T) genotypes, there were significant differences for AUC_0-t_, AUC_0-∞_, Cl/F, T_1/2_ and Ke values (*P* were 0.027, 0.024, 0.024, 0.027, and 0.041, respectively). Third, the presence of the wild-type allele, combination (C/C + C/T), had a significant influence on all pharmacokinetics parameters (*P* values: C_max_ = 0.050, AUC_0-t_ = 0.026, AUC_0-∞_ = 0.016, Cl/F = 0.016, T_1/2_ = 0.004, and Ke = 0.007). *GSTM3*- rs1799735 had a significant effect on AUC_0-∞_ and Cl/F when *A/*A and *A/*B were compared (*P* = 0.041 for both parameters). Regarding *TNF*-rs1800629, the A allele carriers had significantly higher AUC_0-t_, AUC_0-∞_, and T_1/2_ values (*P* = 0.035, 0.030, and 0.025, respectively) and lower Cl/F and Ke values (*P* = 0.030 and 0.025). The homozygous variant (A/A) carrier was not found. MDR1 (*ABCB1*) and OATP1B1 (*SLCO1B1*) were the only ATV transport-related genes with an effect on ATV pharmacokinetics. The variant allele (T) of *MDR1*-rs1045642 produced a significant increase of C_max_ (*P* = 0.037) when the combination of the heterozygous and homozygous variant (C/T + T/T) is compared with the homozygous wild-type (C/C). The C allele of the *SLCO1B1*-rs4149056 polymorphism significantly affected AUC_0-t_, AUC_0-∞_, and Cl/F values (*P* = 0.004 for all three parameters) in homozygous wild-type allele and heterozygous (C/C + C/T) carriers. None of the other 24 polymorphisms tested had a significant impact on ATV pharmacokinetics. The influences of polymorphisms on the ATV pharmacokinetics are shown in Table 3.

**Table 3:** Polymorphisms and genotype clusters with significant effect on ATV pharmacokinetics

		Pharmacokinetics parameters					
Genotypes	N	C_max_ (ng/mL)	AUC_0-t_ (ng/mL*h)	AUC_0-∞_ (ng/mL*h)	Cl/F (L/h)	T_1/2_ (h)	Ke
MTHFR rs1801133							
C/C	14	60.46 ± 20.60	195.77 ± 91.84	213.25 ± 94.16	448.86 ± 202.58	12.95 ± 9.30	0.07 ± 0.04
C/T	37	44.81 ± 24.32*	152.83 ± 87.67	166.43 ± 86.52	595.20 ± 274.47	10.78 ± 5.84	0.08 ± 0.04
T/T	9	34.89 ± 12.90**	141.64 ± 66.29	151.57 ± 67.28	623.70 ± 287.68	9.94 ± 4.19	0.08 ± 0.03
C/T + T/T	46	42.87 ± 22.77^§^	150.64 ± 83.37^§^	163.52 ± 82.64^§^	600.77 ± 274.06^§^	10.62 ± 5.52	0.08 ± 0.03
DRD3 rs6280							
C/C	17	39.90 ± 14.47	149.50 ± 59.86	159.43 ± 57.99	562.72 ± 200.54	8.63 ± 2.33^¢^	0.08 ± 0.02^¢^
C/T	26	44.12 ± 23.77	143.60 ± 85.50	156.77 ± 86.61	654.34 ± 316.38	10.67 ± 6.65	0.09 ± 0.04
T/T	17	58.41 ± 26.70^#^	199.72 ± 102.65^§§, #^	218.89 ± 100.70^§§, #^	431.78 ± 177.83^§§, #^	14.44 ± 8.15^§§, #^	0.06 ± 0.03^§§, #^
C/C + C/T	43	42.45 ± 20.51	145.93 ± 75.66	157.83 ± 75.82	618.12 ± 277.41	9.86 ± 5.43	0.09 ± 0.04
GSTM3 rs1799735							
*A/*A	55	44.71 ± 21.36	158.00 ± 89.57	171.60 ± 90.03	582.27 ± 270.90	10.92 ± 6.24	0.08 ± 0.03
*A/*B	5	71.92 ± 32.15	196.08 ± 33.24	213.93 ± 28.26^¥^	378.87 ± 46.77^¥^	13.82 ± 10.17	0.08 ± 0.07
TNF rs1800629							
G/G	51	45.54 ± 22.80	153.18 ± 82.16	167.14 ± 83.12	586.66 ± 266.42	10.59 ± 6.14	0.08 ± 0.04
G/A	9	55.88 ± 26.94	215.85 ± 96.68^€^	230.57 ± 93.37^€^	391.55 ± 132.91^€^	14.66 ± 8.45^€^	0.06 ± 0.02^€^
MDR1 rs1045642							
C/C	13	33.43 ± 13.40	135.68 ± 78.72	150.53 ± 79.10	699.77 ± 376.59	10.87 ± 4.99	0.08 ± 0.04
C/T + T/T	47	50.78 ± 24.21^¤^	168.22 ± 88.36	181.93 ± 88.95	528.14 ± 216.66	11.24 ± 7.00	0.08 ± 0.03
SLCO1B1 rs4149056							
C/C + C/T	11	53.92 ± 24.72	222.27 ± 91.48	241.23 ± 92.98	390.51 ± 195.46	12.42 ± 8.59	0.07 ± 0.03
T/T	49	45.41 ± 23.01	147.45 ± 80.40^≠^	160.29 ± 79.55^≠^	604.57 ± 264.87^≠^	10.88 ± 6.12	0.08 ± 0.04
*P*-values of genotype combinations on ATV pharmacokinetics							
Clusters		C_max_	AUC_0-t_	AUC_0-∞_	Cl/F	T_1/2_	Ke
A *vs*. B		0.163	0.060	0.041^£^	0.041^£^	0.519	0.519
B *vs*. C		0.007^£^	4 × 10^−4£^	0.001^£^	0.001^£^	0.177	0.177
A *vs*. C		0.016^£^	0.011^£^	0.011^£^	0.011^£^	0.181	0.181
C *vs*. A + B		0.001^£^	9.1 × 10^−5£^	1.31 × 10^−4£^	9.5 × 10^−5£^	0.112	0.399
D *vs*. E		0.187	0.060	0.041^£^	0.002^£^	0.610	0.486
E *vs*. F		2.3 × 10^−4£^	0.001^£^	0.001^£^	0.004^£^	0.241	0.260
D *vs*. F		1 × 10^−3£^	0.014^£^	0.011^£^	6 × 10^−6£^	0.230	0.177
F *vs*. D + E		5.3 × 10^−5£^	3.61 × 10^−4£^	4.22 × 10^−4£^	1.35 × 10^−4£^	0.115	0.176

Data presented as mean ± standard deviation

*P = 0.018 (C/T *vs*. C/C), **P = 0.004 (T/T *vs*. C/C), ^§^ *P* ≤ 0.050 (C/T + T/T *vs*. C/C), ^§§^ *P* ≤ 0.041 (T/T *vs*. C/T), ^¢^ *P* ≤ 0.008 (C/C *vs*. T/T), ^#^ *P* ≤ 0.050 (T/T *vs*. C/C + C/T), ^¥^ *P* = 0.041 (*A/*B *vs*. *A/*A), ^€^ *P* ≤ 0.035 (G/A *vs*. G/G), ^¤^ *P* = 0.037 (C/T + T/T *vs*. C/C), ^≠^ *P* = 0.004 (T/T *vs*. C/C + C/T), ^£^ = significant. Clusters are explained in the main text

### Association between genotypes and metabolizer phenotypes

No individual genotype correlated with any metabolizer phenotype after Bonferroni’s correction for multiple testing. However, MDR1-rs1045642 behavior was remarkable in this aspect, as no homozygous wild-type (C/C) was a slow metabolizer.

Of the six polymorphisms with an effect on ATV pharmacokinetics, three polymorphisms associated with the slow metabolizer phenotype considering genetic models. The C/T or T/T genotype of *MTHFR*-rs1801133, the T/T genotype of *DRD3*-rs6280, and the C/T or T/T genotype of *MDR1*-rs1045642 were significantly associated with slow metabolizer phenotype using dominant, recessive, and dominant models, respectively. This association remained statistically significant after adjusting for multiple testing using Bonferroni’s correction (*P* < 0.05; Table 4).

**Table 4:** Association values between genotypes and metabolizer phenotypes

Association values between genotypes and the slow metabolizer phenotype using dominant, recessive and additive models					
Gene	Polymorphism	Model	OR (95 % CI)	*P*-Value	Pc Value
MTHFR	rs1801133	Dominant (C/C *vs*. C/T + T/T)	C/T + T/T = 0.64 (0.42–0.99)*	0.011	0.028**
			C/C = 3.11 (1.26–7.68)*		
DRD3	rs6280	Recessive (C/C + C/T *vs*. T/T)	C/C + C/T = 0.62 (0.38–1.00)	0.015	0.034**
			T /T = 2.63 (1.21–5.70)*		
GSTM3	rs1799735	Additive (*A/*A *vs*. *A/*B)	*A/*A = 0.88 (0.70–1.09)	0.126	0.308
			*A/*B = 3.50 (0.64–19.20)		
TNF	rs1800629	Additive (G/G *vs*. G/A)	G/G = 0.89 (0.68–1.16)	0.324	0.553
			G/A = 1.82 (0.55–6.00)		
MDR1	rs1045642	Dominant (C/C *vs*. C/T + T/T)	C/T + T/T = 1.45 (1.18–1.77)*	0.008	0.020**
			C/C = 0.06 (0.003–1.00)		
SLCO1B1	rs4149056	Recessive (C/C + C/T *vs*. T/T)	C/C + C/T = 1.33 (0.45–3.99)	0.610	0.884
			T/T = 0.93 (0.70–1.23)		

*OR* odds ratio, *CI* confidence interval, *Pc P*-values adjusted by using Bonferroni’s correction for multiple comparisons, − = Not calculated, ** = P ≤ 0.05. Clusters are explained in the main text

Linear regression analysis using aforementioned genetic models confirmed that these five polymorphisms affected the variability of pharmacokinetic parameters of ATV, except for the *TNF*-rs1800629 polymorphism (Additional file 2).

Next, we analyzed genotype combinations of the six polymorphisms that individually had a significant effect on ATV pharmacokinetic parameters (Table 3 and Additional file 3): cluster A (subjects with genotypes related to normal metabolism), cluster B (subjects with only 1 genotype related to decreased metabolism), and cluster C (subjects with 2 or more genotypes related to decreased metabolism). As the *MTHFR*, *MDR1*, and *DRD3* genotypes effected pharmacokinetics most, clusters that only considered these genes were formed: Cluster D (subjects with *MTHFR*, *MDR1* and *DRD3* genotypes related to normal metabolism), cluster E (subjects in which either *MTHFR*, *MDR1* or *DRD3* gentoype related to decreased metabolism), and cluster F (subjects in which all *MTHFR*, *MDR1* and *DRD3* genotypes were related to decreased metabolism). The analysis of genotype combinations revealed that cluster C, *i.e*. subjects with 2 or more genotypes related to decreased metabolism, had a significant higher C_max_ (*P* ≤ 0.016), AUC_0-t_ (*P* ≤ 0.011) and AUC_0-∞_ (*P* = 0.011), but significantly lower Cl/F values (*P* = 0.011) when compared with cluster A and B. The genotype combination analysis limited to *MTHFR*, *MDR1* and *DRD3* showed that cluster F was significantly different from clusters D and E; a higher C_max_ (*P* = 5.3 × 10^−5^), AUC_0-t_ (*P* = 3.61 × 10^−4^), and AUC_0-∞_ = (4.22 × 10^−4^), but lower Cl/F (*P* = 1.35 × 10^−4^). The influences of the clusters on ATV pharmacokinetics are shown in Table 3.

The association analysis between clusters and phenotypes displayed a mutual dependency and association (*P* ≤ 0.05). The C and F cluster were associated with slow metabolizers as shown in Table 5. The stepwise multiple regression analysis showed that the combination of *MTHFR*, *DRD3* and *MDR1* polymorphisms are related to ATV slow metabolizers. The combination of these three polymorphisms contributed to the pharmacokinetic variability prediction with an R^2^ = 0.295, and adjusted R^2^ = 0.257 with a *P* = 2.26 × 10^−4^.

**Table 5:** Association values between genotypes and metabolizer phenotypes

Association between genotype combinations and metabolizer phenotypes				
Clusters	Phenotypes	OR (95 % CI)	*P*-Value	Pc Value
A, B, C	Rapid, Normal, Slow	-	1 × 10^−3^**	3.1 × 10^−3^**
C, A + B	Slow, Normal + Rapid	4.00 (1.39–11.49)*	3.83 × 10^−4^**	1 × 10^−3^**
D, E, F	Rapid, Normal, Slow	-	6.6 × 10^−5^**	1.6 × 10^−3^**
F, D + E	Slow, Normal + Rapid	4.53 (1.62–12.68)*	7 × 10^−6^**	2.9 × 10^−5^**

*OR* odds ratio, *CI* confidence interval, *Pc P*-values adjusted by using Bonferroni’s correction for multiple comparisons, − = Not calculated, ** = P ≤ 0.05. Clusters are explained in the main text

### Adverse effects

ATV was well tolerated by all subjects, since no volunteer showed any adverse effects during and at the end of the pharmacokinetic study. No clinically significant changes from baseline were observed in the physical examination or the ECG during the study, and no clinically significant mean changes from baseline were observed for any laboratory parameters.

## Discussion

Numerous studies have been performed to better characterize the high variability in ATV pharmacokinetic parameters. In this study, the maximum and minimum C_max_ and AUC_0-t_ values differed by approximately 10-folds in 60 healthy volunteers. Other studies even observed 15-folds for the C_max_ and 12-folds for AUC, after a single dosis of ATV [32]. The interindividual variability exists even though the study subjects are under controlled conditions. Actually, the search of prediction biomarkers for disease risk and the response to treatment is an area of research with great activity. However, so far no pharmacogenetic testing in clinical studies have been carried out in Mexicans. To contribute to the identification of the genetic architecture underlying the drug metabolism and response in the Mexican population, we examined the impact of 30 polymorphisms in genes related to drug metabolism and response on ATV pharmacokinetics.

In this study, we propose a novel and simple approach to classify the metabolizer phenotypes from the analysis of pharmacokinetic profiles. This approach uses C_max_, which reflects the absorption rate, and the AUC_0-t_) that reflects the extent of absorption and clearance. We distinguished three different metabolic phenotypes (slow, normal, and rapid) with significant differences for pharmacokinetic parameters (Table 2). The slow phenotype displayed the highest variability in pharmacokinetic parametes, clearly illustrated by the greater dispersion of C_max_-AUC_0-t_ valuesof the slow metabolizers as compared to the normal and rapid ones. T_1/2_ behaves stochastically, and its variance increased with the time, which may be the reason that there is no significant T_1/2_ difference among metabolizer phenotypes. Our phenotype classification is consistent with the one reported by Quing Huang *et al*., who used a pharmacometabonomic approach to classify 48 healthy volunteers as low, medium, and high ATV metabolizers [32]. Slow metabolizers tend to have higher plasma drug levels and to be more susceptible to adverse side effects. On the other hand, rapid metabolizers tend to have lower plasma drug levels, which may explain a poor drug response. Our results support the use of this classification method for *in vivo* studies.

There was a significant effect of six gene polymorphisms on different pharmacokinetics parameters (*P* ≤ 0.05). T allele carriers of *MTHFR*-rs1801133 had a lower C_max_ and AUC but increased Cl/F. This pharmacokinetic profile is consistent with increased clearance activity and a lower ATV concentration in the body, which may lead to a weaker response to ATV.

To our knowledge, our study is the first that reports an effect of the *MTHFR*-rs1801133 polymorphism on statin pharmacokinetics, and is consistent with previous studies on drug response and cardiovascular disease susceptibility. In 2008, Maitland-van der Zee *et al*. found that the *MTHFR*-rs1801133 C/C genotype protects against coronary heart disease in different populations [33]. Another study reported that the C/C genotype protected against cardiovascular disease in a Turkish population under statin therapy [34]. The C allele frequencies reported in both studies [33, 34] are different from the allele frequencies found in the Mexican population. The *MTHFR*-rs1801133 polymorphism (C677T) causes an Ala → Val substitution which decreases enzyme activity leading to increased homocysteine levels. Hyperhomocysteinemia is a known risk factor for cardiovascular disease [34]. How the T variant of *MTHFR*-rs1801133 augments ATV clearance, so that the drug response is diminished, remains to be elucidated.

With respect to the *DRD3*-rs6280 (Ser9Gly) polymorphism, the homozygous variant genotype (T/T) affected the pharmacokinetic parameters consistent with a slow metabolizer phenotype. So far, the influence of *DRD3*-rs6280 on ATV pharmacokinetics has not been reported. *DRD3* encodes a dopamine receptor and is functionally related to reward stimuli and control of movement [35]. *DRD3*-rs6280 polymorphisms have been mainly related to addictive behavior[36, 37] and involuntary movements [38]. The C allele is related to a stronger intracellular response to dopamine [35]. However, another *DRD3* polymorphism (rs1486012) has been associated with a decrease in lopinavir/ritonavir elimination [39].

Subjects that carry the *B allele of *GSTM3*-rs1799735 had a decreased clearance of ATV and therefore an increased AUC_0-∞_. These results are not consistent with the higher detoxification activity associated with the *B allele [40] of this gene which encodes a glutathione S-transferase M3 that conjugates glutathione with substrates like drugs, toxins, and carcinogens. The *B allele represents a 3-bp deletion in intron 6, which generates a recognition sequence for the Ying Yang transcription factor (YY1), and thus alters the gene expression of *GSTM3*. We found no previous report relating this polymorphism to statin metabolism. The reason for the lack of association of the *GSTM3* polymorphism with the metabolic phenotypes may be due to the low frequency of the *B allele in our relatively small study group. *GSTM3*-rs1799735 has been associated with various types of cancers with different effects [40–42]. Allele frequencies found for rs1799735 are similar to those reported by Jain *et al*. [42]. Nevertheless, the homozygous variant (*B/*B) carrier was absent in our sample.

Heterozygous A allele carriers of *TNF*-rs1800629 had pharmacokinetic parameters that were consistent with a diminished clearance of ATV. Nevertheless, their influence on ATV pharmacokinetic variability was not confirmed by linear regression analysis. The rs1800629 (G/A) polymorphism is located in the promoter region of *TNF. TNF* encodes the pro-inflammatory cytokine tumor necrosis factor alpha. The variant A allele increases protein expression; therefore the wild-type G allele has a protective effect by reducing the risk of thrombosis in patients with hemodialysis [43]. It is unknown how *TNF* is involved in ATV metabolism, but there is evidence that ATV may attenuate *TNF* expression [44], which results in a reduction of the inflammatory process. The allele and genotype frequencies in our study group are similar to those of a Turkish population [43].

Previous studies have reported that the *MDR1*-rs1045642 polymorphism affects the response to ATV treatment. However, different studies disagree with respect to which homozygous genotype results in a better response to therapy [45–47]. *MDR1* encodes a P-glycoprotein transporter, that functions as an ATP-dependent efflux pump and thus protects against harmful substances. *MDR1* is widely expressed, for example in the small intestine, the blood–brain barrier, hepatocytes, and kidney proximal tubules [48, 49]. The *MDR1*-rs1045642 (C3435T) polymorphism is located in exon 26 of the *MDR1*/*ABCB1* at a wobble position that does not produce an amino acid change. However, Hoffmeyer *et al*. [48] have reported that subjects homozygous for the variant (T/T) not only had reduced expression of *MDR1* but also higher drug plasma levels as compared to subjects homozygous for the wild-type (C/C) [48]. In our study, carriers of the variant T allele, either homozygous or heterozygous, had a higher C_max_. Similar results were found by Zhou *et al*. [50] and Gonzalez-Vacarezza *et al*. [51] on fluvastatin and quetiapine pharmacokinetics, respectively. The T allele and genotype frequencies found in our study group were similar to those reported in the Lahu population (0.54) [52], the Dutch population (0.52) [53], and in Caucasians (0.53) [54]; however, they differ from those found in the Chilean population (0.34) [16], the African population (0.17), and in African-Americans (0.39) [55].

Similar to *MDR1* polymorphisms, there are conflicting reports about the response and adverse drug reactions to statins in subjects with the *SLCO1B1*-rs4149056 (SLCO1B1*5) polymorphism [47, 56]. *SLCO1B1*, which is highly expressed in the liver, encodes an organic anion influx pump for numerous compounds. The *SLCO1B1*-rs4149056 (c.T521C) polymorphism results in the substitution of alanine for valine at amino acid residue 174, which reduces transport activity and leads to higher circulating statin concentrations that could be responsible for the reported adverse effects [57]. Our results support that rs4149056 affects ATV pharmacokinetics. However, the polymorphism did not associate with metabolic phenotypes. This could be due to the low frequency of C allele carriers in our sample. The variant T allele is the most frequent in Mexican population.

In summary, in our study we found six polymorphisms in different genes that have a significant effect on the pharmacokinetics of ATV. GSTM3 is a phase II metabolizing enzyme, which explains its impact on clearing parameters (Cl/F), and as a consequence AUC. *MDR1* and *SLCO1B1* are transporters, and this function may explain their impacton ATV pharmacokinetic parameters (C_max_, AUC, and Cl/F). However, the significant impact of the *DRD3*, *MTHFR*, and *TNF* polymorphisms on ATV C_max_, AUC’s, Cl/F T_1/2,_ and Ke is achieved by so far unknown mechanisms.

The value of a pharmacokinetic parameter is the end result of a complex ADME process that involves many proteins. Polymorphisms in the different encoding genes may neutralize each other, which makes it highly unlikely that a single polymorphism determines a metabolizer phenotype. However, a slow metabolizer phenotype may be the result of various polymorphisms that reinforce a certain impact. Indeed the accumulation of polymorphisms of the six genes (*MTHFR*, *GSTM3*, *DRD3*, *TNF*, *MDR1*, and *SLCO1B1*) coincided with a shift from rapid metabolizers (no genotypes related to decreased metabolism in cluster A) to slow metabolizers (cluster C, up to six polymorphisms). *MTHFR*, *MDR1* and *DRD3* polymorphisms seem to have a leading impact on metabolizer phenotype, and were sufficient to identify slow metabolizers in our study group. For example, cluster D, comprised of *MTHFR*, *MDR1* and *DRD3* wild-type genotypes, were rapid metabolizers; cluster E is a mixture of the rapid and normal metabolizers, whereas the accumulation of these three polymorphisms (cluster F) is sufficient to identify slow metabolizers. These results were confirmed by association tests with different models, and stepwise multiple regression analysis. The interference of other genes in the A and B clusters may explain the lack of association with the rapid metabolizer phenotype. This can be explained by the complexity of the process that underlies the pharmacokinetic parameters, which involves many genes and other environmental factors. Thus, if a gene variant has a relatively small impact it will not be detected. The distribution plot (Fig. 2b) illustrates that slow metabolizer phenotype accumulates the widest variability of polymorphisms. Although we found six polymorphism with effect on the ATV pharmacokinetics, we know that the variability is not limited to the presence of these six markers, since other polymorphisms with little or moderate influence and not analyzed in this study, could contribute to the observed variability in each metabolic phenotype.

To our knowledge, this is the first report on the use of a massive genotyping tool (microarrays) to associate gene polymorphisms with pharmacokinetic variability of a drug commonly used by the Mexican population. The pharmacokinetic variability of ATV depends on several factors, including genetic factors. We identified six polymorphisms on six different genes that, individually, had an impact on some or all pharmacokinetic parameters. Absence of all polymorphism corresponded to rapid metabolizers, whereas the accumulation of polymorphisms caused a shift to slow metabolizers. However, some shortcomings existed in our study. First, there were insufficient data to correlate the metabolic phenotypes with the ATV response. Second, the number of participants was not large enough to validate our findings. Hence, our findings need to be validated in a larger population with genotype preselection. In order to identify (a set of) candidate predictors for ATV metabolizer phenotype, confirmative studies should be performed that focuse on the identified six polymorphisms. Our results may also be considered for future meta-analysis. The ultimate aim is that a pharmacogenetic analysis of a set of genes can be used to guide a personalized dosage that ensures drug response and prevents adverse drug effects.

## Conclusions

In summary, this pilot study offers a novel, comprehensive approach to understand the genetic contribution to the variability of ATV metabolism in a Mexican population. It enabled the identification of candidate predictive biomarkers for slow ATV metabolizers. The future confirmation of the predictive potential of these candidate genetic biomarkers and their incorporation in routine genotyping tests may optimize ATV efficacy in the clinical practice.

## Additional files

Additional file 1:
**Allele and genotype frequencies of polymorphism in genes involved in drug metabolism and response.** HWE = Hardy-Weinberg equilibrium, P = gene present, A = gene absent, ǂ = genotypes with frequency < 0.01, ¥ = not calculated. (DOCX 33 kb) Additional file 2:
**The** ***P***-**values of results of linear regression for polymorphisms on ATV pharmacokinetic parameters using different genetic models.** a = Dominant (C/C vs. C/T + T/T), b = Recessive (C/C + C/T vs. T/T), c = Additive (*A/*A vs. *A/*B), d = Additive (G/G vs. G/A), e = Additive (G/G vs. G/A), f = Recessive (C/C + C/T vs. T/T), Pc = *P*-values adjusted by using Bonferroni’s correction for multiple comparisons, − = Not significant, * = Significant. (DOC 36 kb) Additional file 3:
**Genotype clusters.** Genotype combinations are classified as follows: altered genotypes are in red and other genotypes are in green; Cluster A:subjects with genotypes related to normal metabolism, cluster B: subjects with only 1 genotype related to decreased metabolism, cluster C: subjects with 2 or more genotypes related to decreased metabolism, cluster D: subjects with *MDR1* and *DRD3* genotypes related to normal metabolism, cluster E: subjects in which either *MDR1* or *DRD3* was altered, and cluster F: subjects in which both *MDR1* and *DRD3* were altered. (TIFF 3555 kb)

## Abbreviations

ATV: Atorvastatin
ADME: Absorption, distribution, metabolism, and excretion processes
BMI: Body mass index
ECG: Electrocardiogram
HWE: Hardy-Weinberg equilibrium
MAF: Minor allele frequency
C_max_: Maximum plasma concentration
AUC_0-t_: Area under the plasma concentration-time curve from time 0 to the time of the last measurement
AUC_0-∞_: Area under the curve from time 0 to the time extrapolated to infinity
Cl/F: Apparent clearance of the fraction dose absorbed
Ke: Elimination rate constant in the terminal phase
T_1/2_: Half-life in the terminal phase of the drug
HCA: Hierarchical cluster analysis
