# Supplementary material for: A pharmacogenetic pilot study reveals MTHFR, DRD3, and MDR1 polymorphisms as biomarker candidates for slow atorvastatin metabolizers
Source: BMC Cancer. 2016 Feb 8;16:74. doi: 10.1186/s12885-016-2062-2 (PMC4746878; doi:10.1186/s12885-016-2062-2)
Supplement: Additional file 1: — Allele and genotype frequencies of polymorphism in genes involved in drug metabolism and response. HWE = Hardy-Weinberg equilibrium, P = gene present, A = gene absent, ǂ = genotypes with frequency < 0.01, ¥ = not calculated. (DOCX 33 kb) [file 12885_2016_2062_MOESM1_ESM.docx]

**Additional file 1.** Allele and genotype frequencies of polymorphisms in genes involved in drug metabolism and response.

| **Number** | **Gene** | **Polymorphism** | **Allele frequency** | | **Genotype frequency** | | | **Χ^2^ HWE** | **HWE**  **P-value** |
| --- | --- | --- | --- | --- | --- | --- | --- | --- | --- |
| 1 | CYP1A1 | Ile462Val | Ile = 0.67 | Val = 0.33 | Ile/Ile = 0.47 | Ile/Val = 0.40 | Val/Val = 0.13 | 0.6 | 0.439 |
| 2 | CYP1A2 | *1, *1F | *1 = 0.23 | *1F = 0.77 | *1/*1 = 0.08 | *1/*1F = 0.30 | *1F/*1F = 0.62 | 1.564 | 0.211 |
| 3 | CYP2B6 | rs3745274 (G516T) | G =0.78 | T = 0.22 | G/G = 0.60 | G/T = 0.37 | T/T = 0.03 | 0.386 | 0.534 |
| 4 | CYP2C19 | *1, *2 | *1 = 0.84 | *2 = 0.16 | *1/*1 = 0.693 | *1/*2 = 0.29 | *2/*2 = .017 | 0.284 | 0.594 |
| 5 | CYP2C8 | *1, *3 | *1 = 0.91 | *3 = 0.09 | *1/*1 = 0.82 | *1/*3 = 0.18 | *3/*3 = 0.0 | 0.538 | 0.463 |
| 6 | CYP2C9 | *1, *3 | *1 = 0.95 | *3 = 0.05 | *1/*1 = 0.9 | *1/*3 = 0.1 | *3/*3 = 0 | 0.169 | 0.681 |
| 7 | CYP2D6 | *1, *2, *4 | *1 = 0.70 | *2 = 0.22 | *1/*1 = 0.48 | *1/*2 = 0.37 | *2/*2 = 0 | 3.403 | 0.333 |
|  |  |  |  | *4 = 0.08 |  | *1/*4 = 0.074 | *2/*4 = .074 |  |  |
| 8 | CYP3A4 | rs2740574 (*1, *B) | *1 = 0.94 | *1B = 0.06 | *1/*1 = 0.88 | *1/*1B = 0.12 | *1B/*1B = 0 | 0.23 | 0.631 |
| 9 | CYP3A5 | *1, *3 | *1 = 0.19 | *3 = .081 | *1/*1 = 0.03 | *1/*3 = 0.31 | *3/*3 = 0.66 | 0.005 | 0.941 |
| 10 | GSTM1 | P/A | P = 0.63 | A = 0.37 |  |  |  |  |  |
| 11 | GSTM3 | rs1799735 (*A, *B) | *A = 0.96 | *B = 0.04 | *A/*A = 0.92 | *A/*B = 0.08 | *B/*B = 0 | 0.113 | 0.736 |
| 12 | GSTP1 | Ile105Val | Ile = 0.48 | Val = 0.52 | Ile/Ile = 0.22 | Ile/Val = 0.53 | Val/Val = 0.25 | 0.276 | 0.599 |
| 13 | GSTT1 | P/A | P = 0.17 | A = 0.83 |  |  |  |  |  |
| 14 | NAT2 | *4,*5B, *5A, *5C, *5D *6A, *7B, | *4 = 0.29 | *5A = 0.05 | *4/*4 = 0.08 | *4/*5B = 0.23 | *5A/*5C = 0.1 | ¥ | |
|  |  |  |  | *5B = 0.29 |  | *4/*5D = 0.06 | ǂ | ¥ | |
|  |  |  |  | *5C = 0.08 |  | *5B/*5B = 0.08 | ǂ | ¥ | |
|  |  |  |  | *6A = 0.07 |  | *5B/*7B = 0.08 | ǂ | ¥ | |
|  |  |  |  | *7B = 0.06 |  | *6B/*6A = 0.08 | ǂ | ¥ | |
| 15 | TPMT | *1, *3 | *1 = 0.99 | *3 = 0.01 | *1/*1 = 0.98 | *1/*3 = 0.02 | *3/*3 = 0 | 0.005 | 0.940 |
| 16 | MDR1 | rs1045642 (C3435T) | C = 0.48 | T = 0.52 | C/C = 0.22 | C/T = 0.53 | T/T = 0.25 | 0.276 | 0.599 |
| 17 | ADRB1 | rs1801253 (Arg389Gly) | Arg = 0.82 | Gly = 0.18 | Arg/Arg = 0.70 | Arg/Gly = 0.25 | Gly/Gly = 0.05 | 1.08 | 0.298 |
| 18 | ADRB2 | rs1042713 (Arg16Gly) | Arg = 0.31 | Gly = 0.69 | Arg/Arg = 0.12 | Arg/Gly = 0.38 | Gly/Gly = 0.5 | 0.615 | 0.432 |
| 19 | AGTR1 | rs5186 (A1166C) | A = 0.72 | C = 0.28 | A/A = 0.54 | A/C = 0.35 | C/C = 0.11 | 0.979 | 0.322 |
| 20 | BDKRB2 | rs1799722 (C58T) | C = 0.62 | T = 0.38 | C/C = 0.38 | C/T = 0.48 | T/T = 0.14 | 0.036 | 0.847 |
| 21 | DRD3 | rs6280 (Ser9Gly) | Gly = 0.50 | Ser = 0.50 | Gly/Gly = 0.28 | Gly/Ser = 0.44 | Ser/Ser = 0.28 | 1.066 | 0.301 |
| 22 | GRIN2B | rs1806201 (C2664T) | C = 0.65 | T = 0.35 | C/C = 0.44 | C/T = 0.42 | T/T = 0.14 | 0.253 | 0.614 |
| 23 | HTR2A | rs6314 (His452Tyr) | His = 0.89 | Tyr = 0.11 | His/His = 0.78 | His/Tyr = 0.22 | Tyr/Tyr = 0 | 0.886 | 0.347 |
|  |  | rs6313 (C102T) | C = 0.61 | T = 0.39 | C/C = 0.38 | C/T = 0.45 | T/T = 0.17 | 0.186 | 0.666 |
| 24 | ADD1 | rs4961 (Gly460Trp) | Gly = 0.83 | Trp = 0.17 | Gly/Gly = 0.67 | Gly/Trp = 0.28 | Trp/Trp = 0.05 | 0.439 | 0.507 |
| 25 | AGT | rs699 (Met235Thr) | Met = 0.30 | Thr = 0.70 | Met/Met = 0.06 | Met/Thr = 0.47 | Thr/Thr = 0.47 | 0.74 | 0.389 |
| 26 | BCHE | rs1799807 (Asp70Gly)  rs1803274 (Ala539Thr) | Asp = 0.99 | Gly = 0.01 | Asp/Asp = 0.98 | Asp/Gly = 0.02 | Gly/Gly = 0 | 0.004 | 0.948 |
|  |  |  | Ala = 0.875 | Thr = 0.125 | Ala/Ala = 0.75 | Ala/Thr = 0.25 | Thr/Thr = 0 | 1.224 | 0.268 |
| 27 | COMT | rs4680 (Val108Met) | Val = 0.61 | Met = 0.39 | Val/Val = 0.37 | Val/Met = 0.48 | Met/Met = 0.15 | 0.012 | 0.911 |
| 28 | DPYD | rs3918290 (IVS14+1G>A) | G = 1.0 | A = 0.0 | G/G = 1.0 | G/A = 0.0 | A/A = 0.0 | 4 E^-5^ | 0.994 |
| 29 | ERCC2 | rs13181 (Lys751Gln) | Lys = 0.75 | Gln = 0.25 | Lys/Lys = 0.57 | Lys/Gln = 0.37 | Gln/Gln = 0.06 | 0.029 | 0.863 |
| 30 | IL10 | rs1800896 (G1082A) | G = 0.31 | A = 0.69 | G/G = 0.15 | G/A = 0.32 | A/A = 0.53 | 3.98 | 0.046 |
| 31 | MTHFR | rs1801133 (C677T) | C = 0.54 | T = 0.46 | C/C = 0.23 | C/T = 0.62 | T/T = 0.15 | 3.512 | 0.060 |
| 32 | TNF | rs1800629 (G308A) | G = 0.92 | A = 0.08 | G/G = 0.84 | G/A = 0.16 | A/A = 0.0 | 0.402 | 0.525 |
| 33 | TYMS | Del/Ins 3´-UTR 6pb | Del = 0.40 | Ins = 0.60 | Del/Del = 0.13 | Del/Ins = 0.53 | Ins/Ins = 0.34 | 0.74 | 0.389 |
| 34 | VKORC1 | rs9923231 (G1639A) | G = 0.53 | A = 0.47 | G/G = 0.28 | G/A = 0.50 | A/A = 0.22 | 0.001 | 0.972 |
| 35 | ABCG2 | rs2231142 (C421A) | G = 0.83 | T = 0.17 | G/G = 0.68 | G/T = 0.30 | T/T = 0.02 | 0.384 | 0.535 |
| 36 | SLCO1B1 | rs4149056 (C521T) | C = 0.10 | T = 0.90 | C/C = 0.02 | C/T = 0.16 | T/T = 0.82 | 0.329 | 0.566 |

HWE = Hardy-Weinberg equilibrium, P = gene present, A = gene absent,ǂ = genotypes with frequency < 0.01, ¥ = not calculated
