# Supplementary material for: A pharmacogenetic pilot study reveals MTHFR, DRD3, and MDR1 polymorphisms as biomarker candidates for slow atorvastatin metabolizers
Source: BMC Cancer. 2016 Feb 8;16:74. doi: 10.1186/s12885-016-2062-2 (PMC4746878; doi:10.1186/s12885-016-2062-2)
Supplement: Additional file 2: — The P-values of results of linear regression for polymorphisms on ATV pharmacokinetic parameters using different genetic models. a = Dominant (C/C vs. C/T + T/T), b = Recessive (C/C + C/T vs. T/T), c = Additive (*A/*A vs. *A/*B), d = Additive (G/G vs. G/A), e = Additive (G/G vs. G/A), f = Recessive (C/C + C/T vs. T/T), Pc = P-values adjusted by using Bonferroni’s correction for multiple comparisons, − = Not significant, * = Significant. (DOC 36 kb) [file 12885_2016_2062_MOESM2_ESM.doc]

**Additional file 2.** The *P*-values of results of linear regression for polymorphisms on ATV pharmacokinetic parameters using different genetic models.

| **Chromosome** | **Gene** | **Polymorphism** | **C_max_** | | **AUC_0-t_** | | **AUC_0-∞_** | | **Cl/F** | | **T_1/2_** | | **Ke** | |
| --- | --- | --- | --- | --- | --- | --- | --- | --- | --- | --- | --- | --- | --- | --- |
|  |  |  | ***P*** | **Pc** | ***P*** | **Pc** | ***P*** | **Pc** | ***P*** | **Pc** | ***P*** | **Pc** | ***P*** | **Pc** |
| 1 | MTHFR | rs1801133^a^ | 0.012 | 0.017* | 0.088 | - | 0.061 | - | 0.060 | - | 0.248 | - | 0.591 | - |
| 3 | DRD3 | rs6280^b^ | 0.016 | 0.025* | 0.029 | - | 0.013 | 0.017* | 0.013 | - | 0.014 | - | 0.007 | 0.008* |
| 1 | GSTM3 | rs1799735^c^ | 0.011 | - | 0.352 | - | 0.303 | - | 0.101 | - | 0.350 | - | 0.702 | - |
| 6 | TNF | rs1800629^d^ | 0.228 | - | 0.045 | - | 0.043 | - | 0.037 | - | 0.089 | - | 0.052 | - |
| 7 | MDR1 | rs1045642^e^ | 0.017 | 0.033* | 0.235 | - | 0.254 | - | 0.038 | - | 0.858 | - | 0.919 | - |
| 12 | SLCO1B1 | rs4149056^f^ | 0.279 | - | 0.009 | - | 0.004 | 0.008* | 0.014 | 0.017* | 0.486 | - | 0.501 | - |

^a^ = Dominant (C/C *vs.* C/T + T/T), ^b^ = Recessive (C/C + C/T *vs.* T/T), ^c^ = Additive (*A/*A *vs.* *A/*B), ^d^ = Additive (G/G *vs.* G/A), ^e^ = Additive (G/G *vs.* G/A), ^f^ = Recessive (C/C + C/T *vs.* T/T), Pc = *P*-values adjusted by using Bonferroni’s correction for multiple comparisons, - = No significant, * = Significant.
