# Supplementary figures and images for: A pharmacogenetic pilot study reveals MTHFR, DRD3, and MDR1 polymorphisms as biomarker candidates for slow atorvastatin metabolizers
Source: BMC Cancer. 2016 Feb 8;16:74. doi: 10.1186/s12885-016-2062-2 (PMC4746878; doi:10.1186/s12885-016-2062-2)

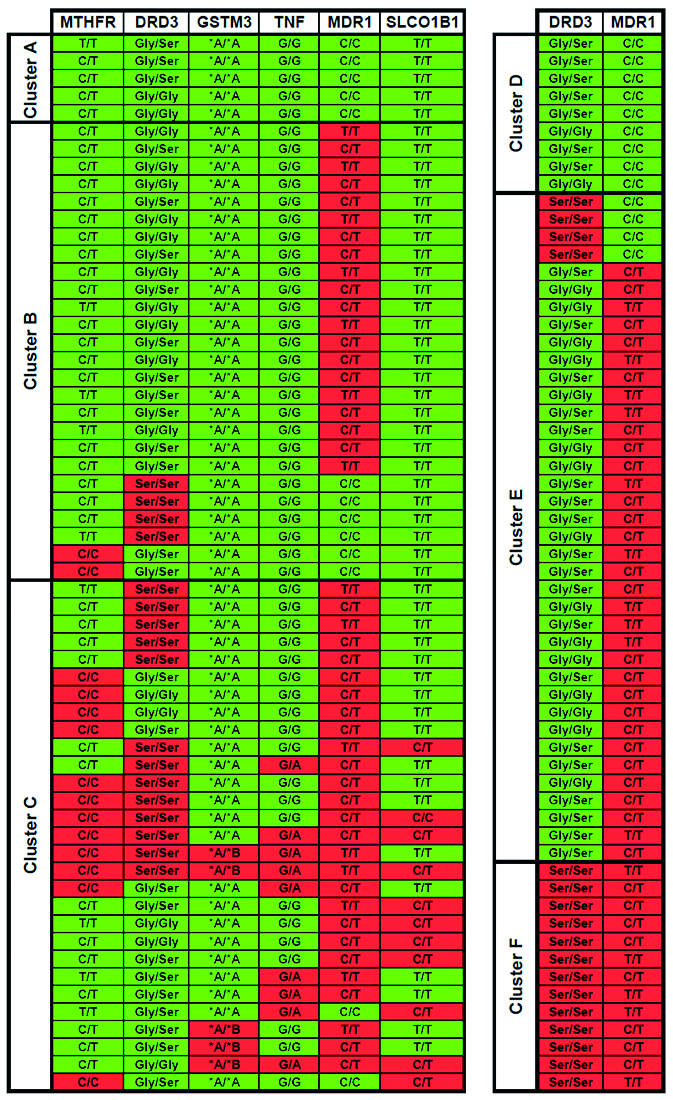

Supplement: Additional file 3: — Genotype clusters. Genotype combinations are classified as follows: altered genotypes are in red and other genotypes are in green; Cluster A:subjects with genotypes related to normal metabolism, cluster B: subjects with only 1 genotype related to decreased metabolism, cluster C: subjects with 2 or more genotypes related to decreased metabolism, cluster D: subjects with MDR1 and DRD3 genotypes related to normal metabolism, cluster E: subjects in which either MDR1 or DRD3 was altered, and cluster F: subjects in which both MDR1 and DRD3 were altered. (TIFF 3555 kb) [file 12885_2016_2062_MOESM3_ESM.tiff]
